# Impact of Prenatal and Postnatal Diagnosis on Parents: Psychosocial and Economic Aspects Related to Congenital Heart Defects in Children

**DOI:** 10.3390/jcm12185773

**Published:** 2023-09-05

**Authors:** Katarzyna Zych-Krekora, Oskar Sylwestrzak, Mariusz Grzesiak, Michał Krekora

**Affiliations:** 1 Department of Perinatology, Obstetrics and Gynecology, Polish Mother’s Memorial Hospital Research Institute in Lodz, 93-338 Lodz, Poland; mariusz.grzesiak@gmail.com; 2 Department of Obstetrics and Gynecology, Polish Mother’s Memorial Hospital Research Institute in Lodz, 93-338 Lodz, Poland; sylwestrzakoskarpatryk@gmail.com (O.S.); krekoram@poczta.onet.pl (M.K.); 3 Department of Prenatal Cardiology, Polish Mother’s Memorial Hospital Research Institute in Lodz, 93-338 Lodz, Poland; 4 Department of Gynecology and Obstetrics, Medical University of Lodz, 90-419 Lodz, Poland

**Keywords:** congenital heart defect, economic, psychology

## Abstract

Congenital heart defects (CHD) are defects detected both prenatally and after birth. They are the most common congenital defects. Despite advances in diagnosis and treatment, CHD remain an important cause of morbidity and mortality in newborns, which has a great impact on economic aspects. It is crucial to provide a holistic approach to the care of children with CHD, including regular cardiac check-ups, appropriate drug treatment, surgical or cardiac interventions as needed, rehabilitation, psychological support, and education for patients and their families. Parents experience a variety of psychological problems. This article summarizes the influence of CHD in the psychological and economic areas.

## 1. Introduction

Congenital heart defects (CHD) are defects frequently detected both prenatally and after birth. They are estimated to occur in about 10 per 1000 live births, with a much higher incidence in premature and stillborn infants [1]. Despite advances in diagnosis and treatment, CHD remain an important cause of morbidity and mortality in newborns. CHD encompass a wide range of defects, from mild lesions that do not cause evident symptoms in childhood and can be incidentally detected in adulthood, to severe anomalies that lead to premature death. There are many factors that increase children’s risk of CHD, which can be divided into genetic and non-genetic [2]. Genetic or environmental causes can be identified in about 30% of CHD cases, and about 17% occur in association with well-defined syndromes such as chromosomal trisomies [3]. There are also environmental factors, such as congenital rubella infection and teratogenic drugs, that have been identified as causes of CHD [2]. Most cases, however, remain unexplained and are likely due to a combination of genetic and environmental factors [4].

CHD result from a complex interplay of genetic and non-genetic factors. Here are a few examples of possible causes of these defects in children:

Genetic Causes of CHD:

Genetic mutations: Congenital heart defects can be caused by mutations in individual genes that regulate heart and blood vessel development.

Genetic syndromes: There are several genetic syndromes, such as Down syndrome (trisomy 21) and Turner syndrome (monosomy X), which increase the risk of congenital heart defects.

Parental inheritance: Sometimes, heart defects can be inherited from generation to generation in a family, suggesting the presence of genetic predisposition.

Non-Genetic Causes of CHD:

Environmental factors: Maternal exposure to harmful chemicals, toxins, alcohol, or smoking during pregnancy can increase the risk of heart defects in the child.

Infections during pregnancy: Certain viral infections, such as rubella and cytomegalovirus, can lead to congenital heart defects if the mother is infected during early pregnancy.

Vitamin deficiencies: Inadequate intake of certain vitamins and minerals, such as folic acid, during the prenatal period can impact heart development in the child.

However, it is important to note that despite identifying genetic and non-genetic causes, it is not possible to explain all cases of CHD. Approximately 70% of congenital heart defects do not have a clearly identified cause, indicating the complex nature of these defects. It is worth emphasizing that research on the causes of CHD is ongoing, and a better understanding of these factors may help identify risk and implement appropriate preventive and therapeutic strategies for children with CHD.

Fetal echocardiography, introduced in the late 1980s, makes it possible to accurately examine the fetal heart. Studies have confirmed the high efficiency of prenatal diagnosis of CHD by experienced specialists [5,6]. Thanks to advances in ultrasound technology, prenatal diagnosis of CHD has reached a high level of accuracy, making it possible to detect almost all forms of CHD before birth as long as echocardiography is performed by highly skilled individuals. Therefore, suspected cardiac abnormalities should always be referred to a fetal cardiology specialist at a tertiary center for further evaluation. The percentage of prenatal diagnoses of CHD increased from 23.0% in 1983–1988 to 47.3% in 1995–2000 [6]. The rate of pregnancy termination following a diagnosis of CHD has also increased.

Regardless of the accuracy of prenatal diagnoses of CHD, the prognosis for children with CHD can vary depending on the type and severity of the defect and the availability and time of appropriate treatment. Prognosis can be difficult to predict for newborns with severe heart defects which require immediate surgical or cardiac intervention. However, thanks to advances in interventional cardiology and cardiothoracic surgery, many heart defects can be successfully treated, improving patients’ prognosis and quality of life.

Children with benign heart defects that do not cause significant symptoms usually have a good prognosis. They often do not require medical intervention and can live normal lives without any limitations. In the case of more complicated heart defects, such as defects associated with the malposition of large vessels or defects associated with valve malformations, the prognosis may depend on the therapy used, the degree of heart damage, and the child’s overall health.

Treatment of CHD may include pharmacotherapy, interventional cardiac procedures (such as closing atrioventricular defects or dilating narrowed blood vessels), or surgical repair of the heart. In some cases, multi-stage corrective surgery may be required, as well as long-term cardiac, physiotherapeutic, and psychological care.

For severe CHD which require complex surgical interventions in the neonatal or early childhood period, the prognosis depends on a number of factors, such as the degree of regurgitation, anatomical complexity, the presence of other concomitant defects, and the child’s overall health. The overall prognosis may be improved by advances in cardiac surgery and intensive care, but still requires long-term monitoring and cardiac care. In this aspect, proper fetal cardiologic management plays a vital role.

It is also important to understand that the prognosis can vary depending on the individual case. Therefore, it is crucial to work closely with a team of specialists, including pediatric cardiologists, cardiac surgeons, neonatologists, and other specialists involved in the treatment of heart defects in children. Prognosis can be improved through regular check-ups, monitoring progress, and implementing appropriate therapies [7,8]. In some cases, further surgical interventions or cardiac procedures may be necessary to improve cardiac function [9].

In recent years, significant progress has been made in the treatment of CHD, allowing many children to achieve good clinical outcomes and lead active lives. However, each case is unique, and the prognosis depends on many factors, such as the type of defect, its severity, the presence of other medical conditions, the child’s general condition, and the quality and availability of medical care. Prenatal diagnosis of CHD causes severe psychological stress for parents. Depression, anxiety, and stress in the mother during pregnancy can negatively affect the newborn’s outcome.

In this research work, the authors utilized a literature review methodology to investigate the impact of prenatal and postnatal diagnosis of CHD on psychosocial and economic aspects related to affected children.

To conduct the review, various keywords were employed to search scientific databases, including “stress”, “depression”, “prenatal diagnosis”, “congenital heart defect”, “fetus”, “parents”, and “treatment costs”.

The authors focused on sources that explored the experiences of parents of children with CHD, both during the prenatal stage and after birth, as well as the implications of these diagnoses on the mental health of parents and children. Additionally, publications discussing the treatment costs and medical care associated with CHD were sought, encompassing expenses related to the treatment of children’s heart defects and the provision of psychosocial support for affected families.

Currently, there is a significant literature gap concerning the specific consequences of children CHD on parents, especially regarding their social life and economic situation. Our aim was to address this gap and provide essential insights on this topic. Additionally, we aimed to identify potential areas with limited research and suggest directions for further studies in this field.

## 2. Psychological Stress

In a study conducted by Brosig et al., higher levels of stress were found in parents of children with a prenatal diagnosis of severe CHD compared to those with a postnatal diagnosis and a control group. Due to the fact that the study was conducted on a very small group of 17 newborns, the reports of the aforementioned researchers should be considered preliminary and require further observation [10]. In another study conducted by Rychik et al., it was observed that partner relationship satisfaction was associated with less maternal stress, and the use of the denial coping mechanism was associated with greater maternal stress, anxiety, and depression [11]. In addition, parents face challenges in adjusting to future life with a child with a congenital heart defect. They may experience concerns about childcare, finances, difficulties in organizing daily life, and emotional strain. Research related to parents’ reactions to a diagnosis of CHD in a child is limited, but there is a growing awareness of the need to better understand this issue. A study by Dagklis and other authors showed that the prevalence of perinatal depression in high-risk pregnancies was as high as 28% [12]. In addition, Woolf-King and colleagues proved that parents of children with severe CHD experience various mental disorders, such as post-traumatic stress disorder, anxiety, depression, stress, and other mental illnesses. Especially the periods before and after children’s surgeries are very stressful [13]. Parents with a prenatal diagnosis may experience greater emotional stress related to the decision to continue the pregnancy, which can lead to long-term stress and negative consequences for both the parents and the child.

Kaugars et al. addressed an important issue related to the impact of prenatal and postnatal diagnosis on the psychosocial and economic aspects of parents of children with CHD [14]. The authors analyzed various aspects of parental functioning, as they have to adjust to the medical and psychosocial demands of their children, both during infancy and throughout childhood. The study confirmed that parents of children with CHD experience more stress, anxiety, and depression compared to the general population. The increasing number of scientific publications highlights the need to pay greater attention to the psychosocial needs of parents of children with CHD. Research has shown that the parents of these children often experience stress related to caregiving, as well as stress related to their child’s illness and developmental disorders. Consequently, such experiences can impact the daily and long-term functioning of the family. It is worth noting that the study included the analysis of various tools to measure parental stress and family quality of life, allowing for a more comprehensive assessment of the situation. It was found that parents of children with more severe forms of CHD experienced greater stress related to the illness and had difficulties coping with this stress. Moreover, parents of children with other coexisting medical problems experienced more stress related to caregiving. The study also had limitations, such as the limited age range of the children studied, which may have affected the generalization of the results. Additionally, the research was primarily based on parental self-reports, which may have introduced certain biases in the results.

Further research in this area is warranted to better understand the psychosocial needs of parents of children with CHD and to tailor appropriate psychological interventions. Effective support and psychological interventions for parents can not only improve their quality of life but also positively impact the health and functioning of their children. Further studies should focus on identifying the factors that influence parental stress to fully integrate psychosocial care for families with children with CHD.

Also, Golfenshtein et al. shed light on the prevalence of CHD as the most common congenital anomaly, affecting approximately one percent of live births [15]. The study highlighted that about 32,000 infants are born with complex, life-threatening CHD conditions in the United States each year, necessitating early surgical interventions and prolonged hospitalizations in the cardiac intensive care unit (CICU). The parents of these infants face increased parenting stress due to several factors, such as the infant’s fragile appearance, illness complications, parenting role alterations in the CICU environment, and long separations from other family members. The study utilized the Parenting Stress Index (PSI) to assess parenting stress in parents of infants with complex CHD and healthy peers during infancy. The PSI measures stressors in various domains related to the child, the parents, and the situation. Parents of infants with complex CHD experience elevated stress levels during critical periods, such as the timing of diagnosis, surgery, CICU stay, and home discharge. Factors such as illness severity, medical procedures, CICU hospitalizations, and infant temperamental and behavioral characteristics contributed to parenting stress in this population. The findings of the study indicate that parents of infants with CHD have significantly higher parenting stress levels compared to parents of healthy infants. The increased stress wasobserved in multiple domains, affecting both parents’ and children’s physical and psychological well-being. Mothers are particularly vulnerable to adverse outcomes during pregnancy, postpartum, and throughout the first year of their infant’s life. It is worth noting that despite the increasing survival rates of complex CHD, parenting stress remains a significant part of daily life for these families, with potential long-term implications. The study calls for longitudinal assessments of parenting stress in this population to better understand how stress evolves over time during the sensitive period of infancy. The results have important implications for healthcare professionals, as identifying especially stressful periods during infancy can help in administering clinical protocols, educating and supporting parents, and designing stress-reducing interventions. By understanding the factors that contribute to parenting stress in this population, healthcare providers can develop targeted strategies to improve the overall well-being of both parents and children with CHD. In conclusion, the study highlights the substantial impact of prenatal and postnatal diagnosis of CHD on various psychosocial and economic aspects for families. It underscores the need for comprehensive support and intervention programs for parents of infants with complex CHD to mitigate the effects of parenting stress and enhance the overall quality of life for these families.

It is also important to consider other psychosocial aspects and quality of life of children with CHD. These children may require emotional and psychological support, as well as access to specialized care for rehabilitation and therapy. Many patients with CHD make significant progress in their development and abilities, but some may experience some limitations.

One potential area for further research could focus on analyzing the long-term effects of prenatal diagnosis of CHD on the psychosocial functioning of parents. Through long-term observations, we could explore how parents cope with the diagnosis and the challenges they face at different stages of their child’s development. Additionally, examining the psychological support and resources that assist parents in coping with difficulties can provide valuable insights for healthcare organizations to tailor their services accordingly. Another intriguing aspect for further research would involve studying the impact of congenital heart defect diagnosis on the siblings of the affected child. Focusing on the psychosocial and emotional aspects of siblings could reveal potential challenges they encounter and the support they may require. Such investigations could aid in understanding the full scope of the diagnosis’s impact on the entire family. Furthermore, while the psychological section of our study presented general challenges faced by parents of children with congenital heart defects, research with a greater focus on individual differences and moderating factors influencing the diagnosis’s impact could provide more precise information. Analyzing the influence of factors such as social support, access to information, previous healthcare experiences, and stress levels can contribute to a better understanding of the psychosocial reactions of parents.

## 3. Role of Healthcare Professionals

It is crucial to provide a holistic approach to the care of children with CHD, including regular cardiac check-ups, appropriate drug treatment, surgical or cardiac interventions as needed, rehabilitation, psychological support, and education for patients and their families.

For each child with CHD, the prognosis can be evaluated individually by a team of medical specialists. It is important for parents and caregivers to maintain regular contact with medical care, ask about any concerns or fears, and follow the recommendations and treatment plan. Prognosis can be improved through regular check-ups, monitoring progress, and implementing appropriate therapies. In some cases, further surgical interventions or cardiac procedures may be necessary to improve cardiac function [1,2].

The psychosocial aspects and quality of life of children with CHD should be considered. These children may require emotional and psychological support, as well as access to specialized care for rehabilitation and therapy [6]. Many patients with CHD make significant progress in their development and abilities, but some may experience some limitations [7].

For each child with CHD, the prognosis can be evaluated individually by a team of medical specialists. It is important for parents and caregivers to maintain regular contact with medical care, ask about any concerns or fears, and follow the recommendations and treatment plan [14].

Throughout the process of treating heart defects in children, it is important to monitor progress and respond to any complications. Regular diagnostic tests, such as echocardiography, electrocardiography, and cardiac function tests, are used to assess the condition of the heart and the effectiveness of therapy [15].

Often, children with CHD require long-term drug treatment. Medications may be used to control symptoms, improve cardiac function, and prevent infections or complications. The type and dosage of drugs are tailored individually to each patient, taking into account the nature of the heart defect and the patient’s overall health [16,17].

In some cases, when drug therapy and surgical interventions are insufficiently successful, heart transplantation may be necessary in children. Heart transplantation is a complex procedure that requires careful evaluation of the patient’s condition and appropriate donor matching [18,19].

The care of a child with a CHD should continue throughout life. Adults who have been treated for a CHD in childhood require regular cardiac follow-up, monitoring for possible complications, and long-term health care management [20].

## 4. Education

Education of patients and their families is also an important aspect of caring for children with congenital heart defects. Patients and their families should be well informed about the nature of the heart defect, treatment, healthy lifestyle recommendations, and ways to cope with possible emotional and psychological difficulties [21,22].

Treatment of CHD in children is a multi-step process that requires comprehensive medical care. Regular appointments, appropriate drug treatment, surgical interventions, rehabilitation, psychological support, and education are key to improving the prognosis and quality of life of children with CHD [23]. In each individual case, the prognosis may vary, so it is important for patients and their families to maintain a close working relationship with their medical team and follow treatment recommendations.

In order to improve the quality of care for children with CHD, there are many organizations and institutions that work to support patients and their families. Examples of such organizations include congenital heart defect foundations, patient associations, and medical centers specializing in the care of these patients [24,25].

In recent years, new technologies and innovations have also emerged to improve the treatment of CHD in children. For example, the development of minimally invasive procedures, such as cardiac interventions, enable effective therapy with less risk of complications and shorter recovery times [26,27]. That could lead to the minimalization of psychological harm.

In addition, advances in genetics and molecular biology are contributing to a better understanding of the genetic basis of CHD and opening up new therapeutic perspectives, such as gene therapy and cell therapy [28,29].

It is also critical to conduct scientific and clinical research to expand our knowledge of congenital heart defects, identify risk factors, and improve diagnostic and therapeutic methods [30,31,32]. This allows for continuous improvement and refinement of patient care.

Finally, public awareness of CHD in children is also an important issue. Improving education and information for the public can contribute to the earlier detection of heart defects, appropriate action, and support for patients and their families [33].

## 5. Financial Issue

In the case of heart defects, the cost of treatment depends on many factors, such as the complexity of the defect, the type of medical procedures, the length of hospitalization, and the therapeutic needs after treatment. Consequently, cost analysis is an important consideration for both families, who face financial challenges, and medical institutions, which need to manage resources effectively. In the United States, hospitalizations of children and adolescents aged 0–20 years with congenital heart disease (CHD) accounted for 3.7% of all hospitalizations in this age range [34]. However, the costs associated with these hospitalizations exceeded USD 5.6 billion in 2009, accounting for 15.1% of the total costs of all hospitalizations for children and adolescents of this age [34].

For children and adolescents with CHD, 26.7% of the costs resulted from hospitalizations of patients with a diagnosis of critical congenital heart disease (CCHD) [1]. The highest hospitalization costs were for patients who were less than a year old, had CCHD (compared to other types of CHD), or died during their hospital stay [34]. The highest costs were for patients with CCHDs such as hypoplastic left heart syndrome, coarctation of the aorta, and tetralogy of Fallot [34].

Although infants under one year of age with CHD generated the highest hospitalization costs, these costs were still significant across all age groups, with the average hospitalization cost among patients aged 1–20 years being USD 25,000 [34]. Despite advances in the detection and treatment of congenital heart defects, there is still little knowledge about the health needs of children and adolescents with CHD and the impact of this population on hospital resource utilization and associated costs [34].

The researchers conducted their study using the 2009 Kids’ Inpatient Database (KID), provided by the Agency for Healthcare Research and Quality’s (AHRQ) Healthcare Cost and Utilization Project (HCUP) [35]. Data from multiple states were included in the analysis, providing a more comprehensive understanding of hospital resource utilization across the US [35].

In the analysis of hospital discharges, the researchers included patients aged 20 years or less who were admitted to the hospital during 2009. All patients had at least one confirmed congenital heart defect and were divided into groups with CCHD and non-critical CHD, as well as into age groups of less than one year, 1–10 years, and 11–20 years. A comparison of hospitalization costs was conducted between patients with CHD and those without the defect, as well as between patients with CCHD and those with non-critical CHD. Hospitalization costs reflect the expenses incurred by a hospital to provide medical services and are not equivalent to what an insurance company or patient sees on an invoice [11,35].

One area for further research could involve analyzing the long-term healthcare costs associated with CHD. Such research could include an assessment of the long-term costs of hospitalizations, cardiothoracic surgeries, pharmacological therapies, and rehabilitation for children with heart defects. Additionally, exploring factors influencing the rise in healthcare costs, such as complications and comorbidities, would be beneficial. Another vital aspect for investigation would be the economic impact on the family of a child with a congenital heart defect. Focusing on an analysis of family expenses related to caring for the child could provide insights into how the diagnosis affects their financial situation and identify potential support mechanisms to assist them in managing the costs. Furthermore, studying the cost-effectiveness of different healthcare models for children with congenital heart defects can provide valuable information for healthcare organizations seeking to optimize costs while ensuring high-quality care. Comparing the costs and outcomes of various treatment approaches can aid in selecting the most effective strategies.

## 6. Conclusions

The treatment of CHD in children is a complex process that requires multiple medical disciplines and an interdisciplinary approach. Regular check-ups, individualized treatment, psychological support, and education are critical to improving the prognosis and quality of life of patients. The pursuit of continuous improvement in medical care, technological innovation, research, and public awareness are key elements in the fight against congenital heart defects in children [3].

It should be emphasized that access to quality medical care and therapy for children with CHD is a significant challenge, especially in regions with weak healthcare systems. In some countries, there is a lack of specialized centers and appropriately qualified medical personnel, which hinders effective patient care [36,37]. In such cases, it is important to take action at the governmental and international level to improve medical infrastructure, train staff, and ensure access to necessary resources and medicines.

In the context of the global development of CHD care, there is a need for international cooperation and knowledge exchange. Organizations such as the International Society for Congenital Heart Disease and other scientific, medical, and charitable institutions are involved in promoting cooperation, organizing conferences, training and educational programs for medical personnel, and exchanging experiences [38,39].

The psychosocial aspects associated with CHD should not be overlooked. Both patients and their families often experience high levels of stress and emotional burden. Psychological support, support groups, and educational programs for families can help improve quality of life and adapt to the challenges of the disease [40,41].

In summary, the care of children with CHD is a complex task that requires the involvement of many players, including medical personnel, charitable organizations, scientific researchers, the public, and government authorities. The pursuit of continuous improvement in medical care, the development of new technologies, scientific research, and public education is key to improving the treatment outcomes and quality of life for patients with congenital heart defects.

Important heart defects generating the highest hospitalization costs are hypoplastic left heart syndrome, coarctation of the aorta, and tetralogy of Fallot. Despite advances in the detection and treatment of CHD, there is a need for more knowledge about the health needs of children and adolescents with heart defects and the impact of this population on hospital resources and associated costs. A study using the KID database may provide important information on hospital resource utilization across the United States.

Further research and analysis are needed to better understand the health needs of this population and develop strategies to optimize medical care while ensuring adequate hospital resources and cost control. Improving the detection, treatment, and management of patients with CHD can help improve the health outcomes and quality of life for children and adolescents with this congenital heart defect.

## Data Availability

Data are available after reasonable request.
